# Association of Genetic Variation at *AQP4* Locus with Vascular Depression

**DOI:** 10.3390/biom8040164

**Published:** 2018-12-05

**Authors:** Anna L. Westermair, Matthias Munz, Anja Schaich, Stefan Nitsche, Bastian Willenborg, Loreto M. Muñoz Venegas, Christina Willenborg, Heribert Schunkert, Ulrich Schweiger, Jeanette Erdmann

**Affiliations:** 1 Department of Psychiatry and Psychotherapy, University Medical Center Schleswig-Holstein—Campus Lübeck, 23562 Lübeck, Germany; anna.westermair@uksh.de (A.L.W.); anja.schaich@uksh.de (A.S.); bastian.willenborg@me.com (B.W.); ulrich.schweiger@uksh.de (U.S.); 2 Institute for Cardiogenetics, University of Lübeck, 23562 Lübeck, Germany; m.munz@uni-luebeck.de (M.M); stefan.nitsche.gloy@gmx.de (S.N.); maria.munozvenegas@uni-luebeck.de (L.M.M.V.); christina.willenborg@web.de (C.W.); 3 DZHK (German Research Centre for Cardiovascular Research), partner site Hamburg/Lübeck/Kiel, 23562 Lübeck, Germany; 4 University Heart Center Lübeck, 23562 Lübeck, Germany; 5 Department of Periodontology and Synoptic Dentistry, Institute of Health, Institute for Dental and Craniofacial Sciences, Charité–University Medicine Berlin, corporate member of Freie Universität Berlin, Humboldt-Universität zu Berlin, 10117 Berlin, Germany; 6 German Heart Center Munich, Technical University Munich, 80636 Munich, Germany; siebe@dhm.mhn.de; 7 DZHK (German Centre for Cardiovascular Research), partner site Munich Heart Alliance, 80636 Munich, Germany

**Keywords:** genome-wide association study, coronary artery disease, late-onset depression, vascular depression, aquaporin, AQP4, chromosome 18q11.2

## Abstract

Despite its substantial clinical importance, specific genetic variants associated with depression have not yet been identified. We sought to identify genetic variants associated with depression by (a) focusing on a more homogenous subsample (vascular depression) and (b) applying a three-stage approach. First, we contacted 730 participants with a confirmed atherosclerotic disease (coronary artery disease) from a population-based study population (German Myocardial Infarction Family Study IV) for psychiatric assessment with the Mini International Neuropsychiatric Interview. Second, we genotyped these patients using genome-wide single nucleotide polymorphism (SNP) arrays. Third, we characterized the SNP via in-silico analysis. The final sample consisted of 342 patients (78.3% male, age = 63.2 ± 9.9 years), 22.8% with a severe depressive disorder. Variant rs528732638 on chromosome 18q11.2 was a genome-wide significant variant and was associated with 3.6-fold increase in the odds of lifetime depression. The locus belongs to a linkage disequilibrium block showing expression quantitative trait loci effects on three putative *cis*-regulated genes, including the aquaporin 4 (*AQP4*) locus. *AQP4* is already known to mediate the formation of ischemic edema in the brain and heart, increasing the size and extent of resulting lesions. Our findings indicate that *AQP4* may also play a role in the etiopathology of vascular depression.

## 1. Introduction

Unipolar depression is a common disorder, with 150 million men and women affected worldwide. With 65.5 million disability-adjusted life years (DALYs) worldwide, it is also one of the leading causes of loss of healthy life years [1]. Regarding the etiology, a bio-psycho-social model is assumed, with an estimated heritability of 35% [2]. However, no major gene locus has been consistently shown to be associated with unipolar depression [3,4,5,6], which may be attributable to a β error due to sample heterogeneity. Thus, research focusing on a more homogenous subsample holds promise, such as analyzing subjects with vascular depression. The vascular depression concept evolved at the end of the last century based on consistent differences between unipolar depression manifesting before or after the age of 50 (early onset depression (EOD) or late onset depression (LOD)) [7,8]. Regarding the clinical presentation, patients with LOD tend to exhibit more substantial psychomotor retardation and executive dysfunction but a less depressed affect than patients with EOD [9,10] and tend to respond poorly to treatment [11,12].

The term “vascular depression” places cerebrovascular lesions at the heart of the etiopathology of LOD. This assumption is based on morphological, functional, and epidemiological data: patients with LOD show greater carotid pathology [13] and more cerebrovascular lesions (such as white matter hyperintensities, silent brain infarctions, and microbleeds) [14,15] than patients with EOD. Additionally, cerebrovascular lesions are associated with a higher prevalence of depressive episodes [16]. The relevance of these cerebrovascular lesions is supported by functional imaging studies showing altered connectivity in patients with LOD, which led to the disconnectivity hypothesis of vascular depression [10,17,18,19]. Furthermore, epidemiological studies link unipolar depression with vascular risk factors [20] and with vascular diseases such as dementia, stroke, myocardial infarction and coronary artery disease (CAD) [21,22,23]. The prevalence of vascular depression in the elderly is estimated to range from 2.4 to 3.4% [24,25]. Regarding the genetics of vascular depression, the only study published to date failed to identify an association with the *SLC6A4* locus of the serotonin transporter gene [26]. Therefore, we conducted a genome-wide association study (GWAS) of severe depressive disorders in patients with a confirmed atherosclerotic disorder, namely coronary artery disease.

## 2. Materials and Methods

### 2.1. Study Protocol and Study Subjects

**Figure 1:**
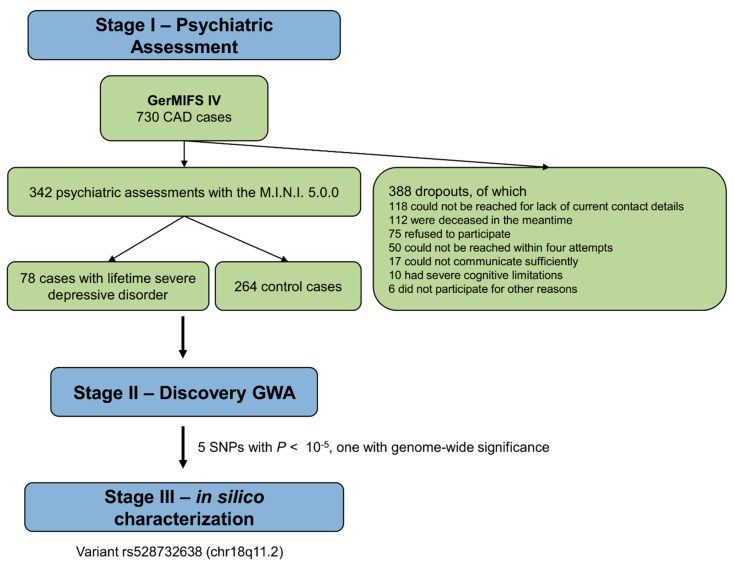
Graphical depiction of the stepwise study design. GerMIFS: German Myocardial Infarction Study; CAD: coronary artery disease; M.I.N.I.: Mini International Neuropsychiatric Interview; severe depressive disorder: severe Major Depressive Episode and/or Dysthymia; GWA: genome-wide association; chr: chromosome; SNP: single nucleotide polymorphism.

The study protocol was approved by the Ethics Committee of the University of Lübeck (ID 04/041) and is depicted in Figure 1. Regarding our study population, we chose to study patients with CAD which correlates highly with cerebrovascular disease [27,28] and thus constitutes an acceptable surrogate marker. When admitted to the University Hospital of Lübeck, Germany, for cardiac catheterization between February 2004 and December 2012, 730 patients with coronary heart disease (154 females, 576 males; mean age = 59.5 years; standard deviation (SD) = 9.8 years; previously described as the German Myocardial Infarction Family Study IV [29]), provided fully informed consent to the use of their data and genetic material for research purposes and to be contacted for further research purposes, in accordance with the Declaration of Helsinki. Of these individuals, 342 patients agreed to participate in our telephone survey when contacted by phone several years later (median = 1.8 years), between March 2013 and January 2015. Exclusion criteria were a lack of informed consent, an inability to participate in a telephone survey (due to cognitive deficits, hearing impairment, aphasia, or insufficient language skills) and the exclusion of CAD during cardiac catheterization.

### 2.2. Assessment of Depression

To further reduce the risk of a β error, we focused on severe depressive disorders (Major Depressive Episode and Dysthymia) which were diagnosed during the telephone survey using the Mini International Neuropsychiatric Interview (M.I.N.I. 5.0.0) [30]. The M.I.N.I. is a short, structured diagnostic interview for mental disorders according to Diagnostic and Statistical Manual of Mental Disorders (DSM) IV with good psychometric qualities. As the timing of the interview was arbitrary, current severe depressive disorders were not differentiated from previous, currently remitted disorders. A detailed report on the psychiatric morbidity found in our sample has been published elsewhere [31].

### 2.3. DNA Isolation and Genotyping

DNA was isolated at the Institute for Cardiogenetics at the University of Lübeck according to standard protocols. Genotyping was performed on a Affymetrix Genome-Wide Human SNP Array 6.0 (Thermo Fisher Scientific, Santa Clara, CA 95051, USA) in cooperation with the Helmholtz-Zentrum in Munich, Germany.

### 2.4. Pre/Post-Processing and Test of Genome-Wide Associations

In our analysis, we used variants that were previously imputed into the 1000 Genomes Phase 3 reference panel. Variants with poor imputation quality (i.e., info < 0.8), a minor allele frequency (MAF) < 0.05 and failing the Hardy–Weinberg-Equilibrium (*p* < 10^−4^) were filtered out. We defined cases as patients with CAD who were diagnosed with severe depressive disorder in the interview (regardless of remission status) and controls as patients with CAD for whom a lifetime diagnosis of severe depressive disorder had been excluded in the interview. Associations were tested with SNPTEST (Department of Statistics, University of Oxford, Oxford, UK) using the additive model [32]. Subsequently, variants with an association level of *p* < 10^−5^ were clustered into independent loci. Loci with at least two variants displaying *p* < 10^−5^ were retained.

### 2.5. Functional Annotation and Software

**Figure 2:**
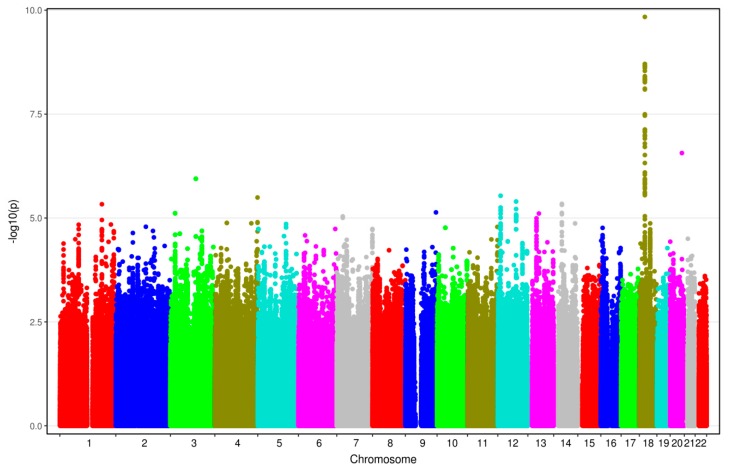
The Manhattan plot shows association levels of severe depressive disorders in patients with coronary artery disease on the autosomal chromosomes.

**Figure 3:**
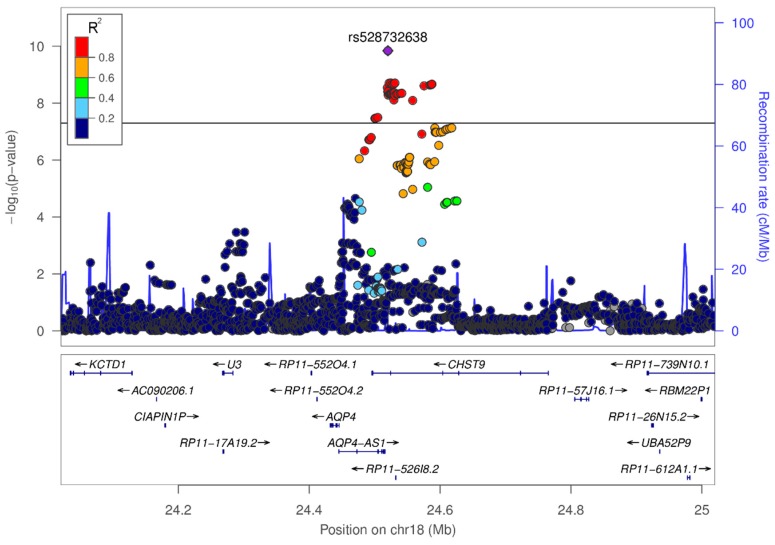
Variant rs528732638 on chromosome 18q11.2 reached genome-wide significance.

Statistical analyses of demographic and clinical characteristics were performed using the Software Package for the Social Sciences (SPSS) (IBM, Armonk, NY, USA). We annotated variants that passed our pre-selected filtering criteria using the Genehopper database (DB) [33]. The Genehopper DB is a relational database that integrates genetic and biomedical data from many public resources. Variants were annotated based on linkage disequilibrium (LD) information [34], expression quantitative trait loci (eQTL) mappings [35,36,37], and topologically associated domain boundaries (TADs) [38]. Figure 1 was created using Powerpoint (Microsoft, Redmond, WA, USA), Figure 2 using the programming language R and Figure 3 with Locuszoom (Department of Biostatistics, University of Michigan, Ann Arbor, MI, USA).

## 3. Results

### 3.1. Demographic and Clinical Characteristics

Of the initial 730 patients, 112 patients were known to be deceased, 75 patients refused to participate, 10 patients were not able to participate due to cognitive limitations, 17 patients were not able to participate because of an inability to communicate (e.g., insufficient language skills, hearing impairment, or aphasia), 50 patients were not able to be reached within four attempts, 118 patients were not able to be reached due to change in contact details, and 6 patients could not be interviewed for other reasons. The final sample consisted of 342 participants (78.3% males; mean age = 63.2 years; SD = 9.9 years) with a lifetime prevalence of severe depressive disorders of 23.0%. The prevalence of severe depressive disorders was higher after manifestation of CAD than before (17.4% vs. 8.8%; McNemar’s χ2 (1) = 11.05; *p* = 0.001), although the corresponding time interval was shorter (median = 7 years vs. median = 54 years; Wilcoxon’s *Z* = −15.36; two-tailed *p* < 0.001). The initial manifestation of 81.0% of severe depressive disorders lay after the initial manifestation of CAD—which was on average at 53.6 years (SD = 9.9 years)—and thus the majority of severe depressive disorders in our sample fit the definition of LOD. 

### 3.2. Results from GWAS

We tested the association of 6,341,066 variants using genotyping data from 78 cases and 264 controls. The genome-wide association levels on the autosomal chromosomes are shown as a Manhattan plot in Figure 2. Five loci passed our pre-defined selection criteria and contained lead variants with *p* < 10^−5^ (Table S1, Figures S2–S5). Among the lead variants, rs528732638 on chromosome 18q11.2 was a genome-wide significant variant (Beta/Se = 1.62; two-tailed *p* = 1.45 × 10^−10^; odds ratio (OR) = 3.62; 95% confidence interval (CI) = 2.38–5.50; Table 1 and Figure 3). Variant rs528732638 is located between the genes *AQP4-AS1* (Aquaporin 4 antisense RNA 1) and *CHST9* (Carbohydrate sulfotransferase 9).

### 3.3. In-Silico Characterization of Variant rs528732638

We next assessed the putative effects of variant rs528732638 and its highly correlated variants (*R*^2^ > 0.8) on gene expression, according to LD information from 1000 Genomes Phase 3 individuals with a European background. Variant rs528732638 shares an LD block with 50 highly correlated variants (Table S2). This LD block showed eQTL effects on five genes in multiple tissues (Table 2 and Table S3) with the eQTL on *AQP4* having the overall lowest *p*-value (*p* = 4.7 × 10^−10^ in blood). We used information about TAD boundaries to group genes into *cis*- and *trans*-located genes with respect to the LD block of rs528732638 (Table S4). Three of the five genes (*AQP4, AQP4-AS1* and *CHST9*) are putative *cis*-regulated genes, i.e., these genes are located within a single spatial compartment in which increased physical interaction occurs, that is between regulatory DNA elements and gene promoters.

## 4. Discussion

### 4.1. Variant rs528732638 on Chromosome 18q11.2 Represents the First Vascular Depression Locus

Variant rs528732638 on chromosome 18q11.2 was associated with a 3.6-fold increase in the odds of lifetime depression in patients with CAD (95% CI = 2.38–5.50). It is the first locus meeting the formal threshold for genome-wide significance [39] of 7.2 × 10^−8^ for an association with depression in patients with coronary artery disease.

Variant rs528732638 is located between the genes *AQP4-AS1* (Aquaporin 4 antisense RNA 1) and *CHST9* (Carbohydrate sulfotransferase 9) and shares an LD block with 50 highly correlated variants. Prior to our study, the GWAS catalog for rs528732638 or one of its LD variants contained no entries [40]. In our study, however, this LD block showed eQTL effects on five genes, three of which (*AQP4*, *AQP4-AS1* and *CHST9*) are putative *cis*-regulated genes. 

*AQP4* is the most abundant aquaporin isoform in the brain, where it is mainly expressed in perivascular astrocyte endfeet [41,42] and in neural stem cells [43,44]. It is a bidirectional water-specific channel that is essential for neurovascular coupling and glymphatic flow [45,46]. A reduced density of AQP4-positive endfeet in the orbitofrontal cortex is associated with major depressive disorder in humans [47] and the antidepressant fluoxetine enhances the endfeet density in an AQP4-dependent manner [48]. Additionally, AQP4 has been shown to play an important role in adult neural stem cell proliferation [49,50], which is downregulated in depression [51]. The antidepressant fluoxetine enhances neurogenesis [52,53,54] in an AQP4-dependent manner [50]. Regarding cerebrovascular disease, abundant evidence suggests a pivotal role for AQP4 in the formation of ischaemic brain edema [55,56,57,58,59,60,61], and AQP4 inhibition reduces infarct volume and improves clinical recovery in an animal model [62,63,64,65]. 

Outside of the central nervous system, *AQP4* is expressed in myocytes in both cardiac and skeletal muscles [66]. Regarding coronary artery disease, AQP4 is involved in cell swelling and cardiac dysfunction in murine myocardial infarction models [67,68]. *AQP4* knock-out in mice reduces the infarct size [69], and tigacelor-mediated cardioprotection relies on *AQP4* downregulation [70]. 

In summary, AQP4 mediates the formation of ischaemic edema in the brain and heart, enhancing cytotoxicity and thus the number and extent of lesions. Based on our finding that variant rs528732638 which belongs to a LD block with eQTL effects on *AQP4* is associated with depression in CAD patients, we speculate that *AQP4* may play a role in the etiopathology of vascular depression via promotion of ischemic cytotoxicity. 

Further studies that aim to replicate our findings in independent, possibly non-European samples, explore expression of *AQP4* in atherosclerosis-relevant tissues as a function of genotype at variant rs528732638, and explore the expression of *AQP4* in depressed patients with and without atherosclerosis, and functional studies (e.g., animal models) are the next potential steps to elucidating the role of *AQP4* in vascular depression.

### 4.2. Limitations of the Study

Nevertheless, limitations of this study must be considered. First, our findings are solely based on a case-control study, not including unbiased prospective population-based cohorts. Second, our sample size is too small to draw definite conclusions. Third, due to the high morbidity in the sample and the length of the follow-up period, our study suffered from a high drop-out rate. As the prognosis of patients with CAD and depression is poorer than that of patients with CAD that are not depressed [71,72,73], selection bias may have occurred, leading to an underestimation of the prevalence of depression and reducing the power of our study. Thus, we may have missed other depression loci in CAD patients. Fourth, we did not diagnose cerebrovascular disease directly using cerebral imaging. Fifth, our finding has not yet been replicated in an independent sample. Sixth, the LD block of the locus we identified also exerts eQTL effects on the *CHST9* gene. Although no previous data have implicated *CHST9* in the etiopathology of vascular depression, we cannot yet exclude this possibility.

## 5. Conclusions

We conducted the first GWAS in a psychiatrically characterized cohort of patients with a confirmed atherosclerotic disorder, namely coronary artery disease. We thus identified a potential locus for vascular depression on chromosome 18q11.2. The lead variant rs528732638 belongs to a LD block with eQTL effects on putative *cis*-regulated genes (*AQP4*, *AQP4-AS1*, and *CHST9*). As *AQP4* is already known to increase the size and extent of ischemic lesions in the heart and brain, we speculate that *AQP4* may play a role in the etiopathology of vascular depression. Replication steps including non-European cohorts are needed to confirm this finding and establish the functional role of the *AQP4* locus in the etiopathology of depression in CAD patients.

## Figures and Tables

**Table 1:** The lead variant of a single locus, rs528732638, showed genome-wide significance.

					EAF			
Variant	Locus	Nearest Genes	EA	NEA	Cas	Con	OR [95% CI]	*P*
rs528732638	18q11.2	*AQP4-AS1, CHST9*	GAA	G	0.34	0.13	3.62 [2.38–5.50]	1.45 × 10^−10^

EA: effect allele; NEA: non-effect allele; EAF: effect allele frequency; Cas: cases; Con: controls; OR: odds ratio; CI: confidence interval; *p* = *p*-value.

**Table 2:** Variant rs528732638 and its high LD variants showed expression quantitative trait loci (eQTL) effects on multiple tissues.

Tissue	Gene
Blood (4.7 × 10^−11^), skeletal muscle (4.7 × 10^−11^)	*AQP4*
Average brain (1.2 × 10^−10^), white matter (6.6 × 10^−9^), parietal lobe (3 × 10^−3^)	*AQP4-AS1*
liver (1 × 10^−6^)	*CDH12* ^a^
Peripheral blood monocytes (1.6 × 10^−6^)	*CHST9*
Peripheral blood monocytes (1.6 × 10^−6^)	*ZNF570* ^a^

^a^*trans*-regulated genes.

## Supplementary Materials

The following are available online at http://www.mdpi.com/2218-273X/8/4/164/s1, Figure S1, Regional association plot of SNP rs11054833 ± 500 kb, Figure S2, Regional association plot of SNP rs11835646 ± 500 kb, Figure S3, Regional association plot of SNP rs7148335 ± 500 kb, Figure S4, Regional association plot of SNP rs60466238 ± 500 kb, Table S1, Lead Variants, Table S2, High LD variants of rs528732638, Table S3, Regulatory effects of rs528732638, Table S4, TAD of rs528732638.
