# Supplementary material for: Association of Genetic Variation at AQP4 Locus with Vascular Depression
Source: Biomolecules. 2018 Dec 5;8(4):164. doi: 10.3390/biom8040164 (PMC6316852; doi:10.3390/biom8040164)
Supplement: Supplementary file 1 [file biomolecules-08-00164-s001.zip › Figures S1-4.docx]

***
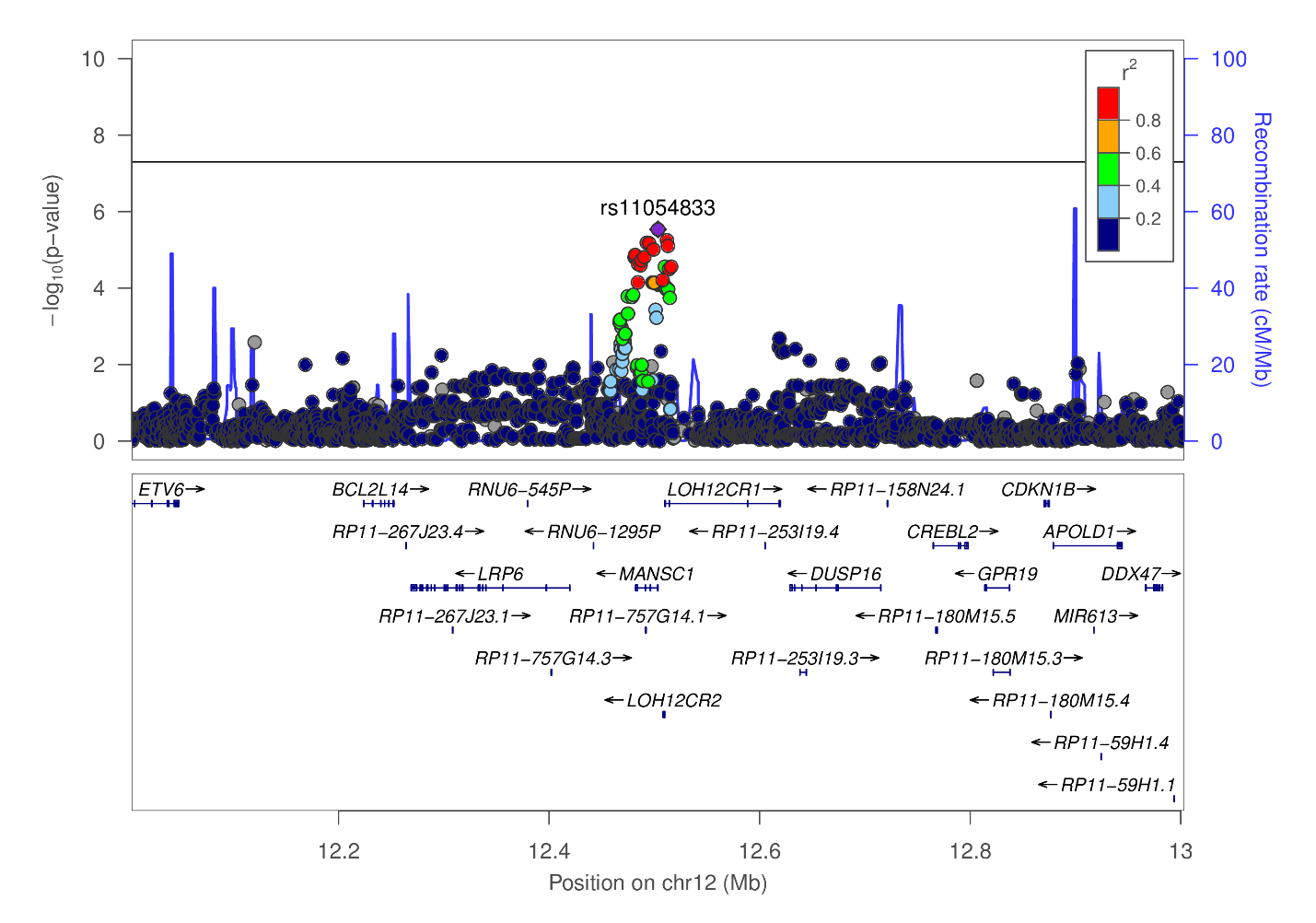
*Figure S1.** Regional association plot of SNP rs11054833 ± 500kb.


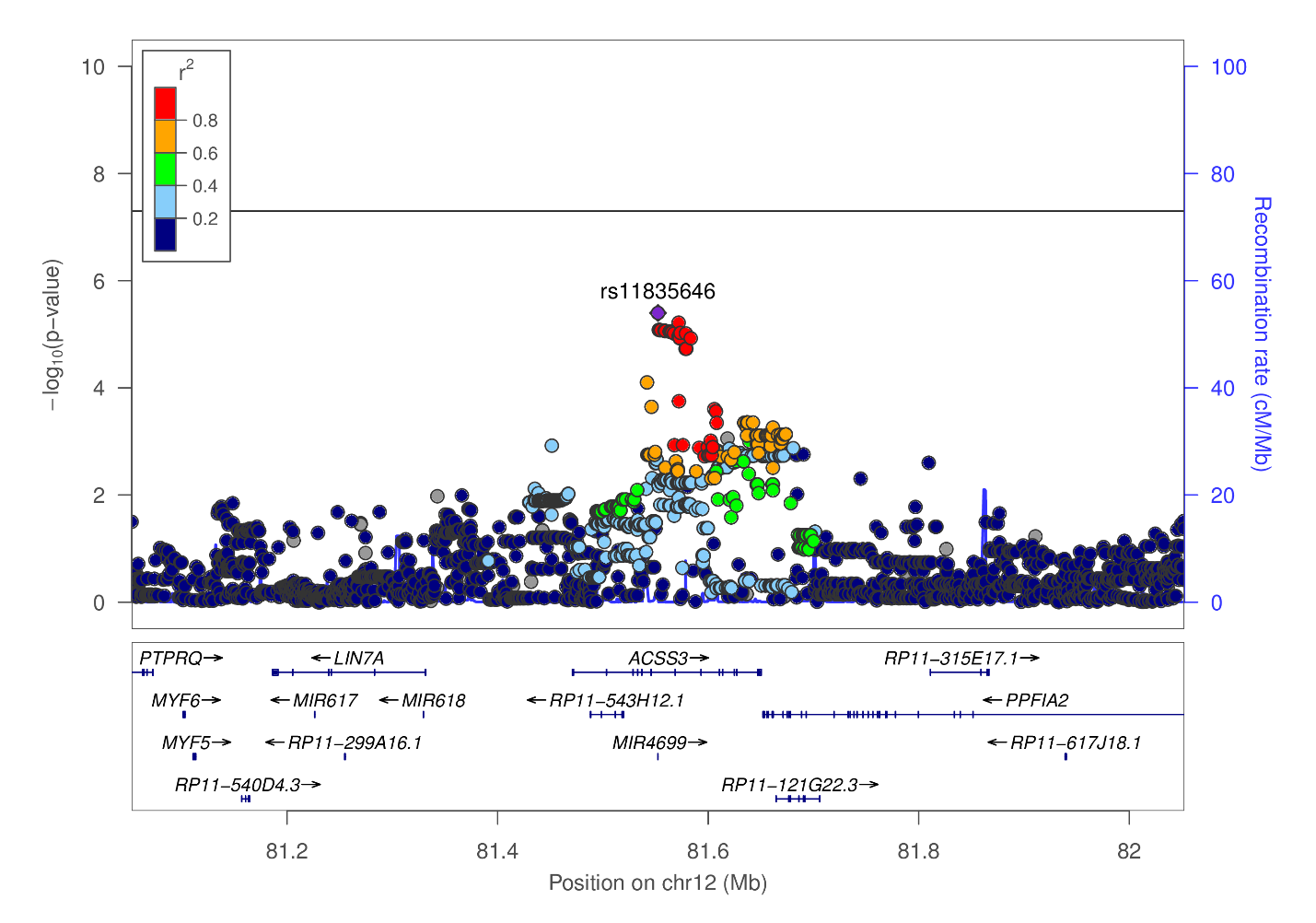


**Figure S2.** Regional association plot of SNP rs11835646 *± 5*00kb.

**
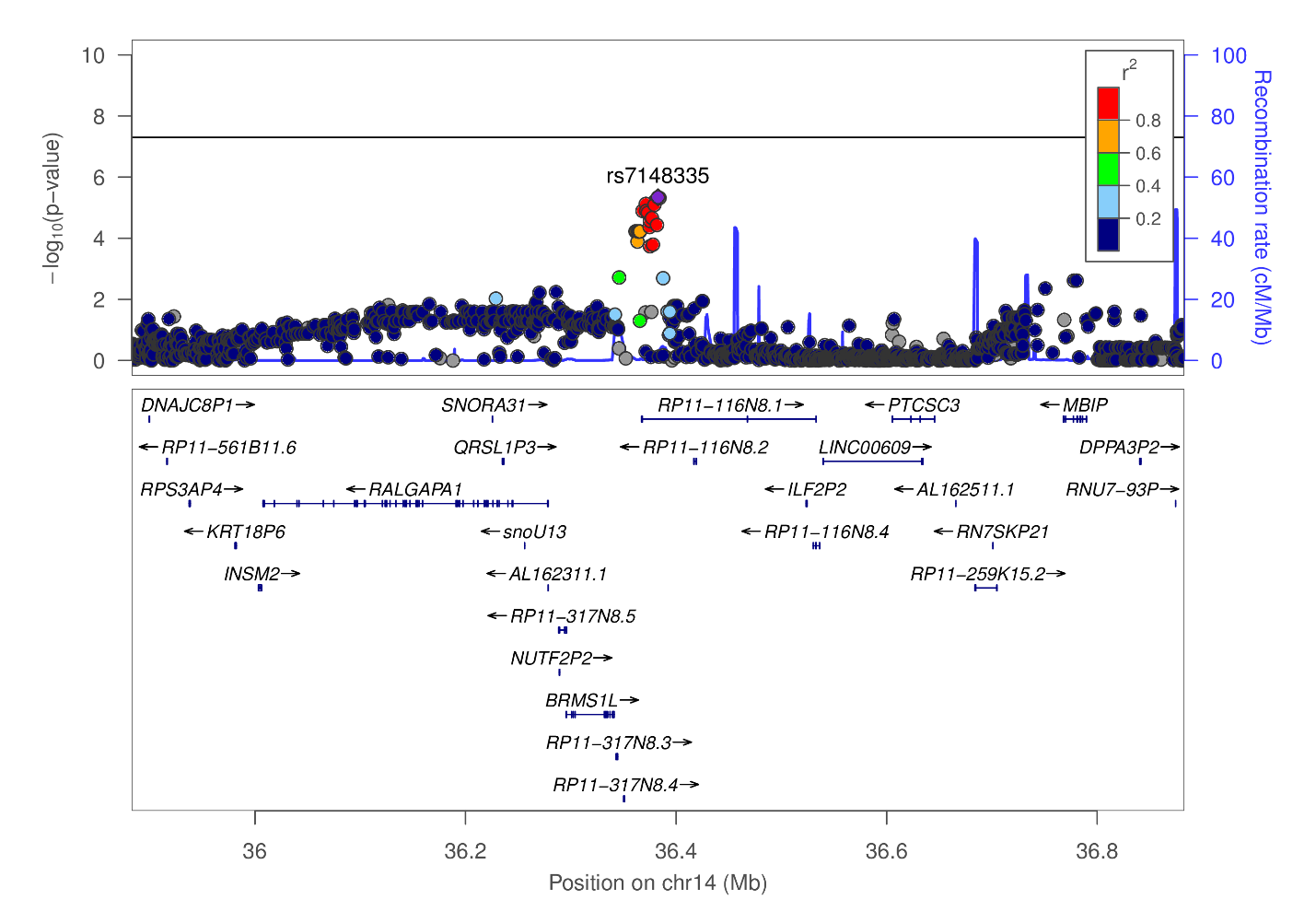
Figure S3.** Regional association plot of SNP rs7148335 *± 5*00kb.

**
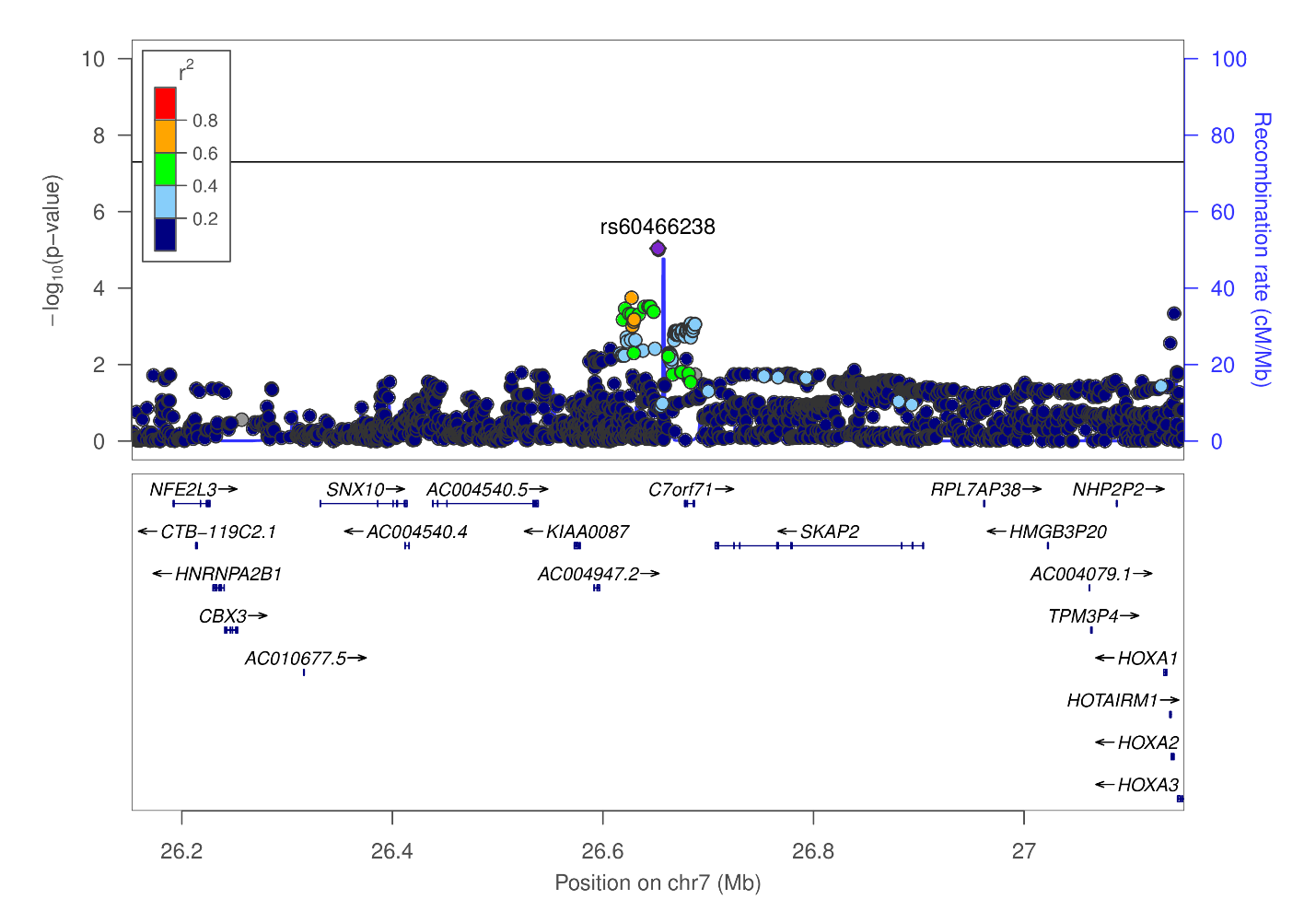
Figure S4.** Regional association plot of SNP rs60466238 *± 5*00kb.
